# The boundaries between PML and PML-IRIS: difficult to define, pathology may predict

**DOI:** 10.3389/fcimb.2025.1607428

**Published:** 2025-06-27

**Authors:** Jiamin Chen, Yanni Du, Lei Sun, Yuyang Dai, Liang Zhang, Xinghuan Ding, Ting Liu, Kun Yang, Xiaoyi Han, Man Li, Xingang Zhou

**Affiliations:** ^1^ Department of Pathology, Beijing Ditan Hospital, Capital Medical University, Beijing, China; ^2^ Department of Radiology, Beijing Ditan Hospital, Capital Medical University, Beijing, China; ^3^ National Institute for Drug Clinical Trial, Beijing Tongren Hospital, Capital Medical University, Beijing, China; ^4^ Department of Neurosurgery, Beijing Ditan Hospital, Capital Medical University, Beijing, China; ^5^ Department of Pathology, Beijing Ditan Hospital, Xuzhou Hospital, Capital Medical University, Xuzhou, China; ^6^ Department of Pathology, Xuzhou Infectious Diseases Hospital, Xuzhou, China

**Keywords:** progressive multifocal leukoencephalopathy, immune reconstitution inflammatory syndrome, aids, pathology, radiology

## Abstract

**Background:**

Progressive multifocal leukoencephalopathy (PML), caused by John Cunningham (JC) virus reactivation, represents a critical neurological complication in AIDS-related immunosuppression. This single-center study conducted a clinicopathological analysis of 19 confirmed PML cases in an AIDS cohort (16 biopsy; 3 surgical specimens), employing comprehensive neuropathological evaluation. Immunohistochemical testing included SV40, NF, NeuN, P53, Ki-67, GFAP, Oligo-2, and CD68. Myelin architecture was evaluated through Luxol fast blue staining, complemented by molecular diagnostics incorporating quantitative JC viral load PCR and metagenomic next-generation sequencing (mNGS).

**Results:**

Notably, 63.2% (12/19) of them had blood CD4+ T-cell counts < 200 cells/μl, and 36.8% (7/19) had ≥ 200 cells/μl. 52.9% (9/17) of the patients had elevated CSF protein, 5.3% (1/19) had decreased CSF glucose. Statistical analysis revealed significant correlations between mass effect and both blood CD4+ T-cell counts (*P* = 0.022) and CSF protein levels (*P* < 0.001). It also demonstrated significant positive correlations between the duration of HIV diagnosis and the degree of inflammatory infiltration (*P* = 0.038) and perivascular inflammatory infiltration (*P* = 0.005), as well as plasma cell infiltration (*P* = 0.011). The degree of inflammatory infiltration was significantly positively correlated with antiretroviral therapy (ART) (*P* = 0.036). The degree of inflammatory infiltration, the presence of plasma cells, and perivascular lymphocytic cuffing were significantly associated with contrast enhancement on imaging studies (*P* = 0.044, *P* = 0.011, and *P* = 0.018, respectively). These cases display characteristics that deviate from the classic PML previously reported, exhibiting a tendency towards MRI enhancement and histologically indicating a more severe inflammatory response, especially for patients following ART treatment.

**Conclusion:**

Our findings suggest that PML and PML-immune reconstitution inflammatory syndrome (IRIS) represent a continuous pathological spectrum, potentially bridged by an intermediate stage with distinct clinicopathological features. This transitional phase may constitute a critical link in the continuum between classic PML and fully developed PML-IRIS. Importantly, it implicates synergistic mechanisms of viral oncogenesis and immune reconstitution, which could redefine therapeutic strategies for this emerging PML variant.

## 1 Introduction

**Figure 1:**
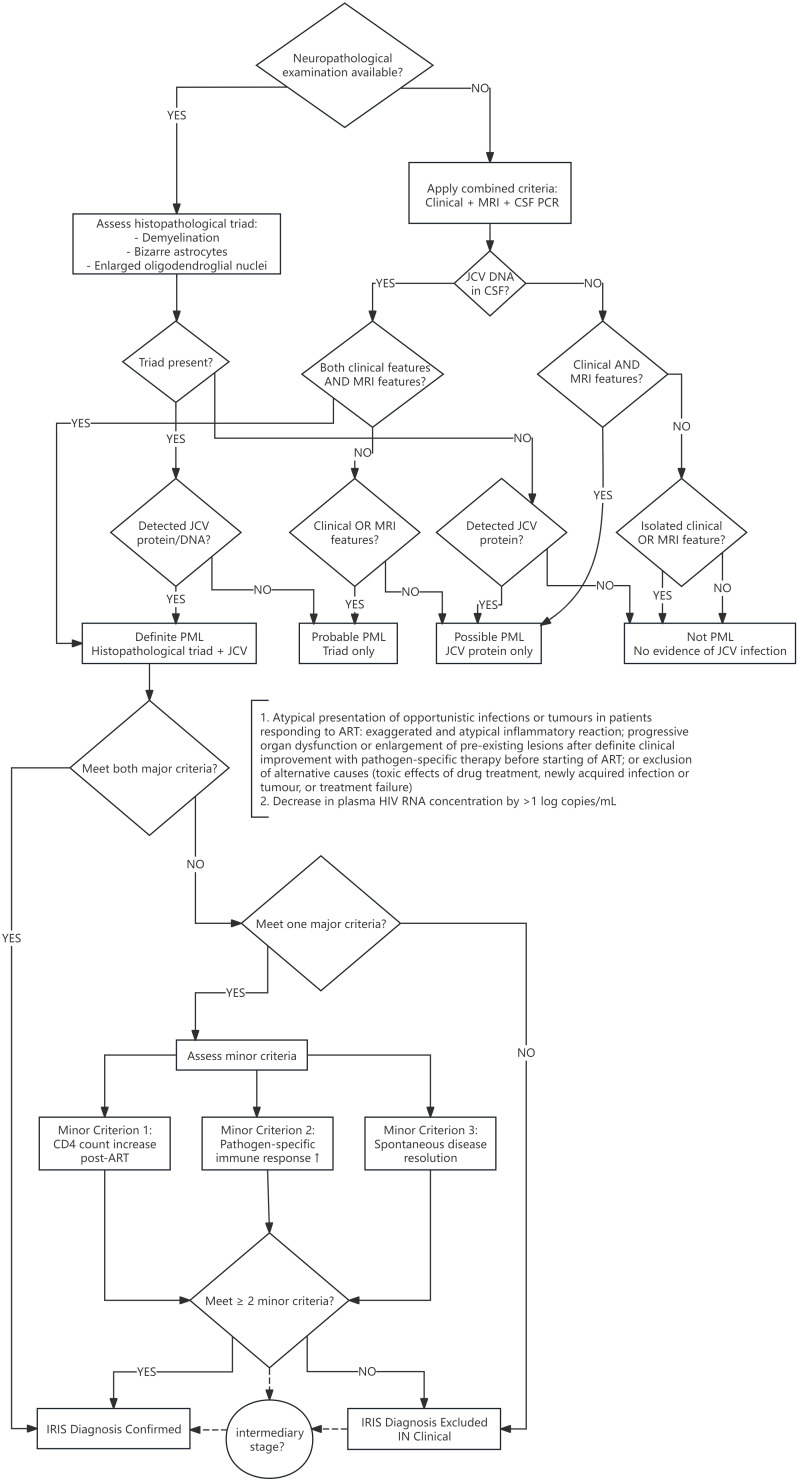
Algorithm for diagnosing PML and IRIS. PML, Progressive multifocal leukoencephalopathy; CSF, cerebrospinal fluid; JCV, John Cunningham virus; ART, active antiretroviral therapy; IRIS, immune reconstitution inflammatory syndrome.

Progressive multifocal leukoencephalopathy (PML) is a viral encephalitis caused by the John Cunningham (JC) virus (Schweitzer et al., 2023). First described in 1958 in patients with hematologic malignancies, PML typically affects individuals with severe immunosuppression. This can result from acquired immune deficiency syndrome (AIDS), hematologic malignancies, solid organ transplantation, or immunosuppressive therapies (White and Khalili, 2011). JCV, an ubiquitous neurotropic polyomavirus, remains latent in the kidneys and bone marrow of healthy individuals (Tan and Koralnik, 2010). PML diagnosis relies on established neuropathological findings or combined clinical, radiological, and laboratory criteria (Berger et al., 2013; Cortese et al., 2021)(**Figure 1**). The disease is characterized by lytic infection of oligodendrocytes, leading to white matter demyelination, primarily in immunocompromised hosts (Gheuens et al., 2013). In human immunodeficiency virus (HIV)-infected patients receiving antiretroviral therapy (ART), immune reconstitution may trigger immune reconstitution inflammatory syndrome (IRIS) in some PML cases. Notably, evolving understanding of disease progression has revealed novel histological characteristics identified during routine diagnostic evaluation.

The objective of this study is to systematically assess the clinical and pathological characteristics of PML within the AIDS patient cohort. Enhancing the depth of knowledge regarding this disease is instrumental in refining diagnostic accuracy for pathologists and in paving the way for innovative approaches in the exploration of underlying mechanisms and the development of therapeutic interventions.

## 2 Materials and methods

### 2.1 Patients and samples

We summarized 19 PML specimens with AIDS confirmed at Beijing Ditan Hospital, Capital Medical University, from June 2021 to December 2024 and analyzed their clinical pathological significance, consisting of 16 biopsy samples and 3 surgical resection samples. This study was in compliance with the Helsinki Declaration and was approved by the ethics committee of Beijing Ditan Hospital (DTEC-KY2024-053-01). Based on the non-interventional nature of this study, informed consent was exempted.

All patients underwent routine preoperative laboratory testing, including blood CD4+ T-cell count, CD4/CD8 T-cell ratio, and blood HIV viral load, cerebrospinal fluid (CSF) protein, CSF sugar and CSF chloride were analyzed (**Supplementary Table 1**).

### 2.2 Immunohistochemistry and Luxol Fast Blue staining

Immunohistochemical testing included SV40 (clone MRQ4), NF (clone 2F11), NeuN (clone A60), P53 (clone DO-7), Ki-67 (clone UMAB107), GFAP (EP13), Oligo-2 (EP112) and CD68 (clone KP1). The IHC primary antibody reagents were purchased from Beijing Zhongshan Jinqiao Biotechnology Co., Ltd. Immunohistochemical staining was performed using a Leica BOND-MAX automated system (Leica Biosystems) according to the manufacturer’s protocols. Both positive and negative controls were incorporated in each experiment (Chen et al., 2023).

LFB staining was employed to demonstrate myelin degeneration, disintegration, or loss. LFB kit was purchased from Wuhan Jinhong Biotech Development Co., Ltd. The detailed experimental procedure was performed as follows: Tissue sections (4 μm) were routinely deparaffinized and hydrated. After a brief rinse in 95% ethanol, the sections were immersed in Luxol Fast Blue (LFB) staining solution and incubated in a 60°C water bath for 2–3 hours (with lid). Subsequently, the sections were removed and excess stain was removed by rinsing in 75% ethanol, followed by a brief wash in running water. Differentiation was performed using lithium carbonate solution for 30–60 seconds until clear distinction between gray and white matter was achieved, followed by another brief wash in running water. Counterstaining was conducted using eosin solution for 10–20 seconds, with a subsequent brief wash in running water. Finally, the sections were routinely dehydrated, cleared, and mounted with mounting medium. Normal myelin sheaths appeared blue-green, while areas of demyelination exhibited red eosinophilic staining.

### 2.3 Real-time polymerase chain reaction and metagenomic next-generation sequencing

DNA extraction: ten sections of FFPE tissues (4μm) were collected into DNase-free tubes for nucleic acid extraction, which was performed using QIAamp DNA FFPE Tissue Kit (56404, US). The extraction protocol involved deparaffinization using xylene and ethanol washes, tissue digestion using incubation with Proteinase K (20mg/mL) at 50°C for ≥ 1 hour (depending on the size of the sections), RNA removal using incubation with RNase A (100mg/mL) at 37°C for 30minutes, DNA recovery using washes and column elutions (30mL elution volume for each block), and quantification of the DNA samples (NanoDrop2000; Thermo Fisher Scientific, Wilmington, DE). Total DNA samples were stored at 20°C for later testing.

JCV Real Time PCR Kit (Beijing GenomePrecision Technology Co., Ltd.) was used for the qualitative detection of fluorescent PCR of JC virus nucleic acid (JCV DNA) in tissue. The real-time PCR was performed using a Roche Z480 system according to the manufacturer’s protocol. The resulting PCR products were separated using agarose gel, purified for cloning, and sent to an external supplier for processing (Genewiz, Tianjin, China).

The mNGS detection process encompasses both experimental operation (wet experiment) and bioinformatics analysis (dry experiment). The wet experiment consists of four steps: sample pretreatment, nucleic acid extraction, library construction, and computer sequencing. The bioinformatics analysis involves the following steps: data quality control, human sequence removal, and identification of microbial species alignment. The entire process was completed by Oumeng V Medical Laboratory (Hangzhou, China).

### 2.4 Statistical analysis

Statistical analyses were performed using SPSS (version 23.0, IBM Inc.). Continuous variables are presented as mean ± standard deviation and compared using Student’s *t*-test or one-way ANOVA. Group differences for categorical variables were assessed using the chi-square test (with Yates’ correction where appropriate) or Fisher’s exact test. Statistical significance was set at *P* < 0.05 for all tests.

## 3 Results

### 3.1 Clinical characteristics

Of the 19 patients, 18 were male, 1 were female. The age at diagnosis was 37.0 ± 11.5 years (16–59 years), and the median age was 36 years.

Clinical manifestations included multiple neuropsychiatric symptoms, 12 patients with motor disorders, 7 patients with visual abnormalities, 7 patients with headache and dizziness, 4 patients with speech disorder, 4 patients with cognitive impairment, and 2 developed fever.

The duration from HIV diagnosis to PML confirmation ranged from 7 days to 5 years, with 6 cases (31.6%) presenting PML as the initial manifestation of HIV infection. Prior to biopsy or surgical intervention, 84.2% (16/19) of the cases had undergone ART.

### 3.2 Laboratory examinations

The CD4+ T-cell counts were 183.3 ± 176.5 cells/μl (19–769 cells/μl), 94.7% (18/19) of patients had lower CD4+ T-cell counts, 63.2% (12/19) of them had blood CD4+ T-cell counts < 200 cells/μl, and 36.8% (7/19) had ≥ 200 cells/μl. The CD8+ T-cell counts were 54.2 ± 430.7 cells/μl (54.2-1611.0 cells/μl), 10.5% (2/19) of patients had lower CD8+ T-cell counts and 31.6% (6/19) of patients had higher CD8+ T-cell counts. The blood HIV viral load test revealed a range of 0–139702 copies/ml, with a median of 465.0 copies/ml. Of the patients, 47.4% (9/19) had HIV counts below 400 copies/ml, while 52.6% (10/19) had HIV counts of 400 copies/ml or higher. 17 patients were tested for CSF protein, sugar, and chloride, and 52.9% (9/17) of the patients had elevated CSF protein, 5.3% (1/19) had decreased CSF glucose. All had normal CSF chloride.

### 3.3 Imaging characteristics

All patients underwent head scans and enhanced magnetic resonance (MR) imaging before surgery. Of the 19 patients, 63.2% (12/19) were located in supratentorial, 10.5% (2/19) were located in infratentorial, and 26.3% (5/19) were located in both supratentorial and infratentorial. The radiological manifestations were characterized by multifocal and asymmetric white matter lesions, exhibiting hypointensity on T1-weighted imaging (T1WI), hyperintensity on T2-weighted imaging (T2WI), and fluid-attenuated inversion recovery (FLAIR) sequences. Mass effect was observed in 21.1% (4/19) of cases, while contrast enhancement was present in 31.6% (6/19) of cases. Restricted diffusion on diffusion-weighted imaging (DWI) was noted in 15.8% (3/19) of cases. Notably, some cases demonstrated characteristic imaging features, including the “Galaxy Sign” or “Shrimp Sign”. Statistical analysis revealed significant correlations between mass effect and both blood CD4+ T-cell counts (*P* = 0.022) and CSF protein levels (*P* < 0.001). Furthermore, contrast enhancement showed significant associations with CSF protein levels (*P* = 0.032) (**Table 1**).

**Table 1:** Clinical and Imaging characteristics of 19 patients with PML.

Patient Characteristic		Mass effect (yes/no)	p value	Contrast enhancement (yes/no)	p value	Restricted diffusion (yes/no)	p value
Age(years)		30.5 ± 16.2/38.7 ± 9.9	0.214	31.3 ± 11.6/39.5 ± 10.9	0.152	53.7 ± 5.0/33.8 ± 9.4	0.003*
Median Age (years)		26.5(16-53)/36.0(26-59)	–	33.5(16-49)/36(26-59)	–	39(49-59)/35.5(16-56)	–
Site	Supratentorial	3/9	0.590	3/7	0.176	2/10	0.685
	Infratentorial	0/2		0/2		0/2	
	Supra& infratentorial	1/4		3/2		1/4	
The duration of HIV diagnosis	≥1month	3/11	1.000	6/8	0.128	2/12	1.000
	<1month	1/4		0/5		1/2	
ART	yes	3/13	0.530	6/10	0.517	2/14	0.422
	no	1/2		0/3		1/2	
blood CD4+ T-cell counts (cells/μl)		357.3 ± 290.8/ 136.9 ± 104.7	0.022*	274.8 ± 261.5/141.0 ± 109.9	0.128	131.3 ± 97.2/193.0 ± 188.3	0.593
blood CD8+ T-cell counts (cells/μl)		921.3 ± 596.3/ 750.5 ± 394.6	0.497	982.5 ± 386.4/696.0 ± 433.5	0.185	530.4 ± 690.9/834.5 ± 392.3	0.274
CD4/CD8 ratio		0.33 ± 0.27/0.20 ± 0.13	0.199	0.32 ± 0.25/0.19 ± 0.11	0.125	0.20 ± 0.14/0.23 ± 0.18	0.812
blood HIV viral load (copies/ml)		23112.8 ± 46058.2/29701.0 ± 45204.1	0.803	69.7 ± 56.6/41349.9 ± 49491.6	0.060	17.1 ± 26.8/8.5 ± 16.4	0.922
CSF protein (mg/dl)		86.8 ± 17.2/42.9 ± 15.0	<0.001**	66.2 ± 25.8/42.2 ± 16.5	0.032*	35.3/51.6 ± 23.2	0.505
CSF glucose (mmol/l)		126.9 ± 1.0/3.1 ± 0.4	0.281	125.3 ± 2.8/42.2 ± 16.5	0.758	2.7/3.2 ± 0.4	0.171

Continuous variables are presented as mean ± SD or median. PML, Progressive multifocal leukoencephalopathy; HIV, human immunodeficiency virus; ART, active antiretroviral therapy; CSF, cerebrospinal fluid.

*p < 0.05. **p < 0.01.

### 3.4 Pathological characteristics

White matter structures were identified in all 19 cases. Among the 3 cerebellar cases, the granular layer was observable. In the 16 supratentorial cases, cerebral cortex was detected in 11 cases. Demyelination was observed in all 19 cases, accompanied by white matter gliosis. The inflammatory infiltration exhibited a spectrum of severity. The 19 cases were categorized as mild, moderate, or severe based on the extent of inflammatory cell infiltration. Mild inflammation was characterized by a small number of inflammatory cells infiltrating the brain tissue, scattered or arranged in small clusters, forming lymphovascular cuffs, but without the cells merging into sheets. Moderate and severe inflammation indicated a greater number of inflammatory cells infiltrating the brain tissue and merging into sheets. Inflammation was classified as moderate when the proportion of inflammatory cells was less than 50%, and as severe when the proportion exceeded 50%. Chronic inflammation was characterized by the infiltration of lymphocytes, plasma cells, and histiocytes. The presence of neutrophil infiltration defined it as acute inflammation. 7 cases demonstrated mild inflammation, while 12 cases presented moderate to severe inflammatory cell infiltration, including 4 cases with prominent inflammatory cell hyperplasia. Chronic inflammatory cell infiltration predominated in 10 cases, while active inflammation was observed in 9 cases. Lymphocytic and histiocytic infiltration was evident in all 19 tissue sections. Notably, 8 cases exhibited significant foamy macrophage (gitter cell) infiltration, 10 cases showed plasma cell infiltration, and 4 cases demonstrated prominent neutrophil infiltration. Eosinophilic intranuclear inclusions were variably present in all cases. Atypical cells were identified in 6 cases. Perivascular inflammatory infiltration was observed in 13 cases, predominantly composed of lymphocytes and plasma cells in 11 cases, with 2 cases showing substantial foamy macrophage infiltration around vessels. Among the 11 cases with identifiable cortical structures, neuronal degeneration was detected in 5 cases, with SV40 immunostaining confirming neuronal involvement. Creutzfeldt cells were observed in 3 cases, including abortive forms, with 1 case displaying pathological mitotic figures.

**Figure 2:**
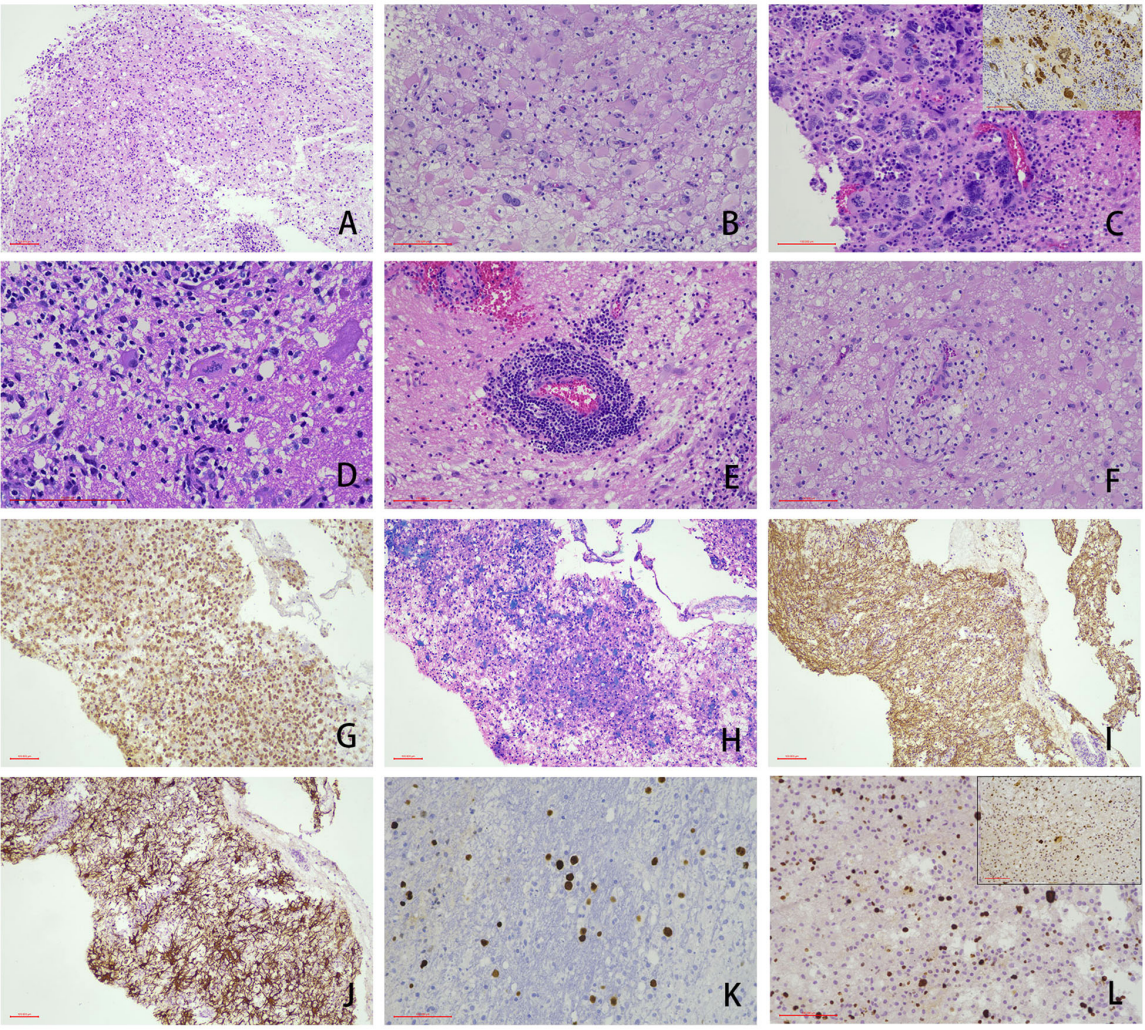
Representative cases showing the typical morphology of PMLs (scale bar: 100μm). Under microscopic examination, the white matter architecture exhibited gliosis accompanied by abundant macrophage infiltration **(A)**. Reactively hyperplastic astrocytes demonstrated enlarged, hyperchromatic nuclei with occasional binucleated cells **(B)**. In specific cases, astrocytes displayed markedly bizarre nuclei (SV40 staining, top right corner) **(C)**. Creutzfeldt cells were observed **(D)**, while perivascular lymphoplasmacytic cuffing **(E)** and perivascular foamy cell cuffing **(F)** were present in some cases. Immunohistochemical analysis revealed: CD68 positivity confirmed extensive histiocytic infiltration **(G)**. LFB+HE staining demonstrated demyelinated lesions with histiocytes phagocytosing myelin debris **(H)**. NF staining showed preserved axonal structures **(I)**. GFAP highlighted reactive astrocytic proliferation **(J)**. SV40-positive nuclear expression was detected **(K)**. Focal positivity for P53 and elevated Ki67 proliferation index were observed in the majority of large cells **(L)**, and this histopathological profile necessitated differential diagnosis from neoplastic lesions.

The absence of LFB staining, coupled with relative preservation of NF immunoreactivity, confirmed demyelination within the brain tissue. CD68-positive macrophages engulfed myelin debris. Oligo-2 immunostaining revealed oligodendrocyte nuclei exhibiting enlargement and hyperchromasia, indicative of JC virus infection; this finding was further corroborated by SV40 nuclear positivity. GFAP immunoreactivity demonstrated reactive astrocytosis. Immunohistochemical analysis confirmed SV40 positivity in all 17 cases examined. Additionally, variant cells displayed positive expression for P53 and Ki67. In the 4 cases showing inflammatory cell hyperplasia, diffuse positivity for both CD4 and CD8 was detected (**Figure 2**).

**Figure 3:**
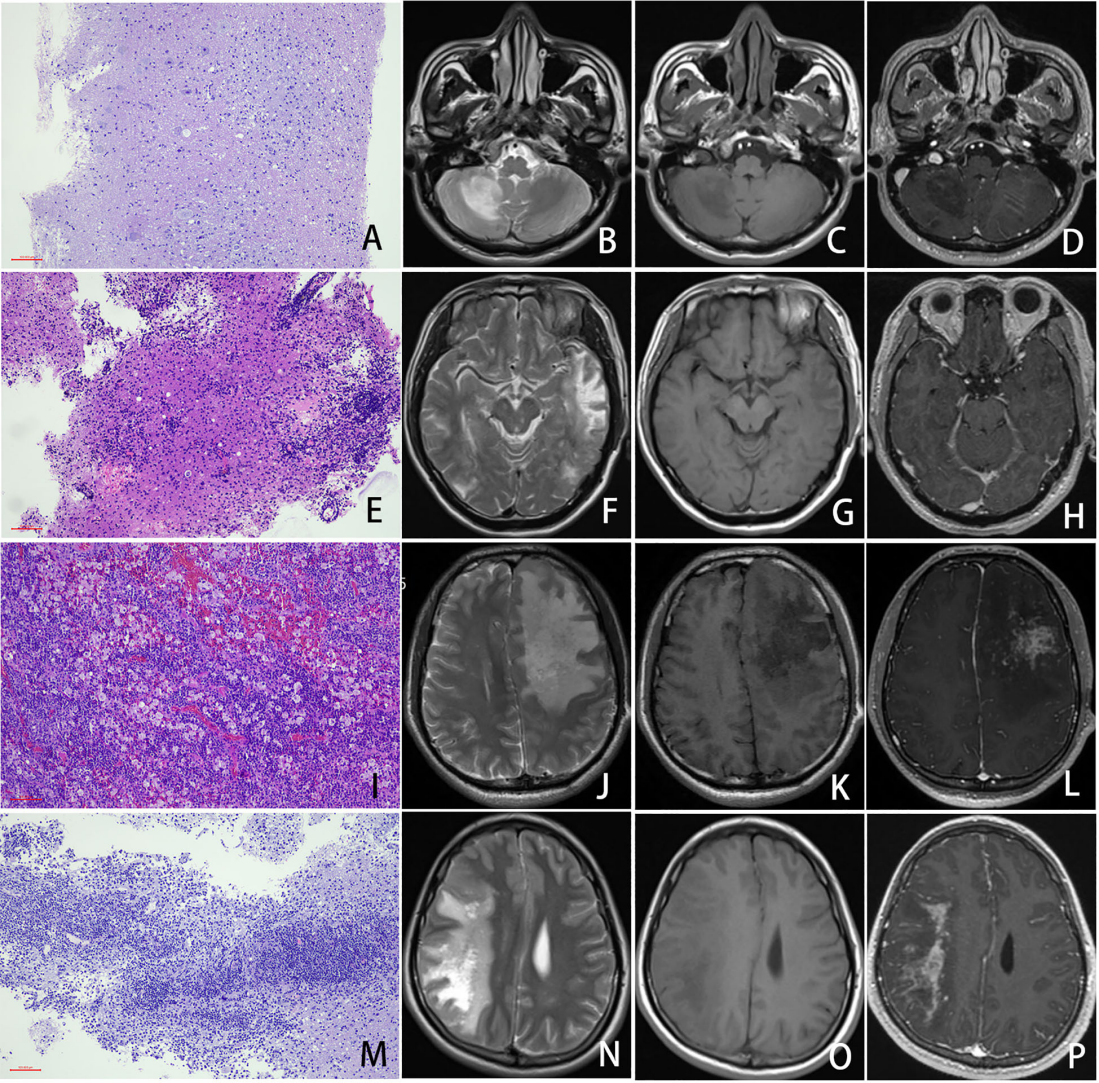
Imaging and Pathological Features of PML. In a PML case with mild inflammation (**(A)** HE staining, 100× magnification, scale bar: 100μm), imaging revealed a patchy hyperintense lesion in the right cerebellar hemisphere on T2-weighted imaging (T2WI) **(B)**, corresponding to patchy hypointensity on T1-weighted imaging (T1WI) **(C)**, with no significant post-contrast enhancement **(D)**. A PML case with moderate inflammation **(E)** demonstrated a larger lesion involving the left temporal and occipital lobes. T2WI showed patchy hyperintensity **(F)**, while T1WI revealed a central hypointense and peripheral isointense pattern **(G)**. Focal mild enhancement was observed in the left temporal lesion post-contrast **(H)**. In a PML case with severe inflammation **(I)**, extensive lesions were noted predominantly in the left frontal lobe, accompanied by mild perilesional edema and a subtle mass effect. The lesions appeared hyperintense on T2WI **(J)**, hypointense on T1WI **(K)**, and exhibited marked patchy enhancement post-contrast **(L)**. A clinically confirmed PML-IRIS case with histopathological evidence of severe inflammation **(M)** showed widespread lesions involving the right cerebral hemisphere, characterized by a prominent mass effect. T2WI demonstrated patchy hyperintensity intermixed with areas of higher signal intensity **(N)**, while T1WI displayed hypointensity **(O)**. Post-contrast imaging revealed pronounced patchy enhancement **(P)**.

Statistical analysis demonstrated significant positive correlations between the duration of HIV diagnosis and the degree of inflammatory infiltration (*P* = 0.038) and perivascular inflammatory infiltration (*P* = 0.005), as well as plasma cell infiltration (*P* = 0.011). Furthermore, the degree of inflammatory infiltration was significantly positively correlated with ART (*P* = 0.036). Additionally, the presence of abundant neutrophils, plasma cells, and perivascular lymphocytic cuffing showed significant positive associations with blood CD8+ T-cell counts (*P* = 0.001, *P* = 0.004, and *P* = 0.012, respectively). Notably, the degree of inflammatory infiltration, the presence of plasma cells, and perivascular lymphocytic cuffing were significantly associated with contrast enhancement on imaging studies (*P* = 0.044, *P* = 0.011, and *P* = 0.018, respectively) (**Figure 3**, **Table 2**; **Supplementary Table 2**).

**Table 2:** Clinical and pathological characteristics of 19 patients with PML.

Patient Characteristic		Degree of inflammation		p value	Perivascular inflammatory infiltration		p value	Neutrophil infiltration		p value	Plasma cell infiltration		p value
		Mild (N=7)	Moderate to severe (N=12)		Lymphocytes and plasma cells (N=11)	Less or gitter cells (N=8)		None or less (N=4)	More (N=15)		Yes (N=10)	No (N=9)	
The duration of HIV diagnosis	≥1 month	3 (15.8%)	11 (57.9%)	0.038*	11 (57.9%)	3 (15.8%)	0.005**	4 (21.1%)	10 (52.6%)	0.530	10 (52.6%)	4 (21.1%)	0.011*
	<1 month	4 (21.1%)	1 (5.3%)		0	5 (26.3%)		0	5 (26.3%)		0	5 (26.3%)	
ART	yes	4 (21.1%)	12 (63.2%)	0.036*	11 (57.9%)	5 (26.3%)	0.058	4 (21.1%)	12	1.000	10 (52.6%)	6 (31.6%)	0.087
	no	3 (15.8%)	0		0	3 (15.8%)		0	3 (15.8%)		0	3 (15.8%)	
blood CD4+ T-cell counts (cells/μl)		57.8 ± 37.3	238.3 ± 197.3	0.074	241.4 ± 197.1	82.2 ± 111.2	0.093	185.8 ± 203.7	183.3 ± 130.0	1.000	249.6 ± 205.7	93.1 ± 105.6	0.084
blood CD8+ T-cell counts (cells/μl)		631.6 ± 241.7	953.3 ± 412.2	0.022*	987.6 ± 401.-	622.3 ± 261.7	0.012*	702.5 ± 290.6	1366.5 ± 187.9	0.001**	1034.0 ± 390.4	608.3 ± 241.7	0.004**
CD4/CD8 ratio		0.11 ± 0.08	0.27 ± 0.19	0.198	0.27 ± 0.19	0.13 ± 0.14	0.204	0.25 ± 0.19	0.13 ± 0.08	0.229	0.27 ± 0.20	0.15 ± 0.14	0.292
Contrast enhancement	yes	0	7 (36.8%)	0.044*	6 (31.6%)	0	0.018*	2 (10.5%)	4 (21.1%)	0.557	6 (31.6%)	0	0.011*
	no	7 (36.8%)	6 (31.6%)		5 (26.3%)	8 (42.1%)		2 (10.5%)	11 (57.9%)		4 (21.1%)	9 (47.4%)	
	no	5 (26.3%)	11 (57.9%)		10 (52.6%)	6 (31.6%)		3 (15.8%)	13 (68.4%)		9 (47.4%)	7 (36.8%)	

Continuous variables are presented as mean ± SD or median. PML, Progressive multifocal leukoencephalopathy; HIV, human immunodeficiency virus; ART, active antiretroviral therapy; CSF, cerebrospinal fluid.*p < 0.05. **p < 0.01. Table presents only statistically significant findings. Non-significant data are not shown for brevity.

### 3.5 PCR and mNGS

JCV Real-Time PCR testing was positive in all 10 cases tested. Among 17 cases analyzed by mNGS, JCV was detected in all cases, with relative abundances ranging from 99.33% to 100%. Analysis focused solely on the primary pathogen, excluding suspected pathogens, suspected background microorganisms, or microorganisms of unclear clinical significance. Of these 17 cases, 13 exhibited single JCV infection. Co-infections were identified in the remaining 4 cases: three with Epstein-Barr virus (EBV) (relative abundance 0.01%-0.18%) and one with Streptococcus pneumoniae (relative abundance 0.67%). One of the EBV co-infected cases also showed co-infection with Mycobacterium avium complex (MAC) (relative abundance 0.01%) (**Supplementary Table 2**).

## 4 Discussion

The JC virus is prevalent in the human body, particularly in the kidney and brain tissues. It seldom causes disease in individuals with normal immune systems; however, it can reactivate in those with compromised immunity (e.g., a CD4+ T lymphocyte count of less than 100 cells/uL) (Brew et al., 2010). HIV and JCV have a synergistic effect that can lead to PML. When AIDS is complicated by central nervous system infections, the blood-brain barrier’s permeability to JCV increases. The HIV Tat protein or chemokines secreted by it can stimulate the replication of JCV (Enam et al., 2004). The infection also affects cerebellar granule cell neurons and cortical pyramidal neurons within the gray matter, resulting in extensive and multi-stage demyelination. As the National Infectious Disease Center (Beijing), our center has treated a large number of HIV patients. As the number of diagnosed cases increases, we have observed numerous new findings in clinical biochemical parameters, imaging features, and pathomorphological characteristics that diverge from those described in traditional textbooks and literature.

Falcó et al. have shown that the occurrence of PML is related to lower blood CD4 levels (Falcó et al., 2008). In our study, we also found that over ninety percent of the cases had low blood CD4 levels, but close to forty percent of the cases had CD4 levels > 200 cells/μl. Furthermore, we also found that over eighty percent of the cases underwent biopsy or surgery after starting ART. This suggests that even in HIV patients with CD4 levels > 200 cells/μl and standardized ART, vigilance for the occurrence of PML is still necessary.

The study of Trunfio has shown that the typical MRI manifestations of PML are usually confined to the white matter, involving the subcortical “U” fibers, with a predilection for the parietal, occipital, and frontal lobes, although they can also occur in the basal ganglia, thalamus, and corpus callosum. Edema and mass effect are uncommon, and lesion enhancement is rare. The lesions are irregular in shape, with low signal intensity on T1WI, high signal intensity on T2WI/2-FIAIR, and no restricted diffusion on DWI, although there have been reports of peripheral enhancement, especially in treated HIV patients (Trunfio et al., 2019). Our study revealed similar imaging findings, with the difference being that approximately 20% of cases exhibited mass effect and about 30% exhibited enhancement. Furthermore, our study found that mass effect was more likely to occur in cases with higher blood CD4+ T-cell counts (*P* = 0.022) and higher CSF protein (*P* < 0.001), while enhancement was more likely to occur in cases with higher CSF protein (*P* = 0.032).

Due to the possibility that patients may be very weak and have a lower resistance, imaging examinations combined with JCV PCR of cerebrospinal fluid are typically used to diagnose PML. These patients avoid invasive brain biopsy. Whiteman showed that before the introduction of ART, PCR for JCV-DNA was 72%-92% sensitive and 92%-100% specific for the diagnosis of PML (Augusto et al., 2015; Whiteman et al., 1993). However, Cinque found that it has become common to have negative CSF molecular results in patients with AIDS with clinical and imaging presentations indistinguishable from those of PML. This may be due to the immune restoration with antiretroviral therapy leading to a decreased viral replication and increased clearance of JCV DNA from the CSF (Cinque et al., 2003). As a result of this, the sensitivity of PCR testing for JCV-DNA is now estimated to be around 58% (Antinori et al., 2003; Marzocchetti et al., 2005). In our group of 19 cases, 6 underwent cerebrospinal fluid NGS testing before surgery, and 50% (3/6) of them detected JCV. For cases where imaging or cerebrospinal fluid etiology cannot be clearly defined, brain biopsy becomes particularly important. These cases with atypical imaging or cerebrospinal fluid pathogens often exhibit more changes in histology.

The study of Berger et al. indicates that the histopathological features of PML are characterized by multifocal demyelinating lesions predominantly affecting the white matter, enlargement of oligodendrocyte nuclei, chromatin with a ground-glass appearance, and increased size of astrocytes with bizarre morphology and irregular, darkly stained nuclei. The inflammatory response is relatively mild, primarily consisting of lymphocytic infiltration, often seen around blood vessels. Neurons are relatively preserved, with lesions primarily affecting the white matter and glial cells, and late-stage tissue necrosis and cavitation may occur (Berger, 2014). All 19 cases in this study were confirmed to have JCV infection through molecular testing (10 by JCV-specific PCR and 17 by mNGS of lesion tissues). Among the 17 cases tested by mNGS, 4 exhibited co-infections. Given the consistently low relative abundance of co-infecting pathogens (0.01%-0.67%), we primarily attribute the pathological changes to JCV infection. Our study indicates that over 60% of cases exhibit moderate to severe inflammation, and nearly half of the cases show active inflammation. Our research also found that cases with higher blood CD8+ T-cell counts are more likely to experience severe inflammatory responses (*P* = 0.022), including a significant number of neutrophils (*P* = 0.001), plasma cell infiltration (*P* = 0.004), and lymphatic vessel cuffing (*P* = 0.012). Cases that have undergone ART were also more likely to experience severe inflammatory responses (*P* = 0.036). Cases with an HIV diagnosis duration exceeding one month were also more likely to experience severe inflammatory responses *(P* = 0.038), significant plasma cell infiltration (*P* = 0.011), and lymphatic vessel cuffing (*P* = 0.005). Cases with enhanced imaging findings were more likely to show moderate to severe inflammation (*P* = 0.044), have plasma cell infiltration (*P* = 0.011) and lymphatic vessel cuffing histologically, rather than gitter cells (*P* = 0.018). These cases display characteristics that deviate from the classic PML previously reported, exhibiting a tendency towards MRI enhancement and histologically indicating a more severe inflammatory response, especially for patients following ART treatment.

Many studies on the immune response against PML have focused on the role of T cells. Patients exhibiting stronger JCV-specific T cell responses tend to have a better prognosis (Khanna et al., 2009). The research of Khanna et al. Showed that survival was correlated to an increase in CD4+ but not in CD8+ JCV-specific T cells. Du and Koralnik et al. showed that within the CNS, however, CD8+ T cells appear to play a critical role. They also proved that the presence of JCV-specific cytotoxic T lymphocytes in the peripheral blood at the time of diagnosis is associated with a better clinical outcome (Du Pasquier et al., 2004; Koralnik et al., 2002; Lima et al., 2007).

Our research results also indicated that cases with higher blood CD8+ T-cell counts are more prone to immune reactions. Furthermore, among the 19 cases studied, 4 exhibited very significant inflammation, with 1 case clinically confirmed to have developed PML-IRIS. Immune reconstitution can be established in HIV patients treated with ART. Forced immune reconstitution frequently may result in profound clinical exacerbation of PML, often associated with high morbidity and mortality. During the immune reconstitution process, cytotoxic T cells eliminate a wide range of cells infected with JCV. Consequently, the excessive immune response exacerbates tissue injury within the lesions (Bauer et al., 2015).

Despite proposed diagnostic criteria (Müller et al., 2010)(**Figure 1**), IRIS manifestations are diverse and lack precise definition (Kaplan et al., 2009), making its diagnosis and the distinction between PML and PML-IRIS clinically challenging. Typically characterized by fever and deterioration of the underlying opportunistic infection, differentiating IRIS from disease progression due to new/worsening infection remains difficult yet critical due to the potential need for risky corticosteroids. Most IRIS cases present within 4–8 weeks of ART initiation in patients with high pre-ART viral loads and low CD4+ counts, though later onset or occurrence in sequestered sites is possible.

In our 12 cases with severe inflammation, the time for ART initiation ranged from 1 to 48 weeks, among which 4 cases with very severe inflammation, the time for ART treatment ranged from 8 to 12 weeks(**Supplementary Table 3**). Our clinically diagnosed case of PML-IRIS underwent 12 weeks of ART exhibited mass effect on imaging, accompanied by patchy enhancement, and histologically there was a significant infiltration of lymphocytes and plasma cells. After corticosteroid treatment, the general condition of this case improved, and the patient was discharged in stable condition. Jan et al. demonstrated that the primary difference between PML and PML-IRIS is the quantity of infiltrating immune T and B cells (Bauer et al., 2015). However, our research suggests that the infiltration of a large number of inflammatory cells is not exclusive to PML-IRIS.

In our cohort of 19 cases, 16 patients with PML received preoperative ART, with durations ranging from 7 days to 1 year. Among these, 4 cases presented with CNS infections as the initial manifestation. Of the remaining 12 cases, 6 exhibited worsening of clinical symptoms 1–3 months after ART initiation. Histopathological examinations revealed prominent inflammatory infiltrates in 4 cases, supporting a diagnosis of PML-IRIS, with one case confirmed clinically. In contrast, 2 cases demonstrated mild histological inflammation, which was insufficient for a PML-IRIS diagnosis, suggesting symptom exacerbation unrelated to immune reconstitution (**Supplementary Table 4**). Our research indicates that PML and PML-IRIS constitute a continuous process with an intermediate link that may exist. Due to the complexity of clinical symptoms and imaging manifestations, neurologists often find it challenging to clearly define these cases, and may even suspect neoplastic lesions. This phenomenon was initially overlooked in our diagnosis, suggesting that clinicians consider other etiological infections. After the mNGS sequencing, it was officially confirmed as a simple JC virus infection.

In clinical practice, an increasing number of PML cases no longer require pathological biopsy to confirm the presence of PML. Pathologists are increasingly encountering so-called “atypical” cases, which also exhibit atypical tissue morphology. These cases present higher demands and challenges for accurate diagnosis by pathologists. Given the rarity of these cases, further data accumulation is necessary for this study. Additionally, there is a scarcity of explorations into the underlying mechanisms.

Our study reports on typical PML cases and also describes a unique category of PML cases characterized by the presence of enhancement and mass effect on MRI, in addition to the classical PML. Histologically, there is a more severe inflammatory response, particularly in patients undergoing ART, where the imaging and pathological morphology overlaps with that of PML-IRIS. The inflammatory response observed histologically occurs before the clinical onset of IRIS, and a definitive diagnosis can alert clinicians to the possibility of IRIS, prompting early intervention and treatment.

## Data Availability

The original contributions presented in the study are included in the article/**Supplementary Material**. Further inquiries can be directed to the corresponding authors.
