# Supplementary material for: The boundaries between PML and PML-IRIS: difficult to define, pathology may predict
Source: Front Cell Infect Microbiol. 2025 Jun 27;15:1607428. doi: 10.3389/fcimb.2025.1607428 (PMC12245874; doi:10.3389/fcimb.2025.1607428)
Supplement: Supplementary file 1 [file Table1.docx]

**Supplementary Table 1. the clinical data of PML**

| **NO.** | **gender** | **Age(years)** | **samples** | **symptom** | **site** | **Mass effect** | **contrast enhancement** | **Restricted diffusion** | **The duration of HIV diagnosis** | **the time of ART** | **blood CD4+ T-cell counts (cells/μl)** | **blood CD8+ T-cell counts (cells/μl)** | **CD4/CD8 ratio** | **blood HIV viral load (copies/ml)** | **CSF protein (mg/dl)** | **CSF glucose (mmol/l)** | **CSF chloride (mmol/l)** | **cerebrospinal fluid mNGS** |
| --- | --- | --- | --- | --- | --- | --- | --- | --- | --- | --- | --- | --- | --- | --- | --- | --- | --- | --- |
| 1 | male | 16 | surgery | motor disorders, speech disorder | both | yes | yes | no | 1 year | 12 months | 769 | 1076 | 0.71 | 93 | high | normal | normal | - |
| 2 | male | 44 | biopsy | motor disorders, speech disorder | supratentorial | no | no | no | 10 months | 10 months | 19 | 326 | 0.06 | 101762 | normal | normal | normal | - |
| 3 | male | 26 | biopsy | motor disorders | supratentorial | no | no | no | 4 years | 1 year | 159 | 524 | 0.30 | 465 | normal | normal | normal | negtive |
| 4 | male | 36 | biopsy | motor disorders | supratentorial | no | no | no | 1 month (initial manifestation) | 7 days | 144 | 1101 | 0.13 | 139702 | high | normal | normal | negtive |
| 5 | male | 35 | biopsy | motor disorders, visual abnormalities | supratentorial | no | no | no | 2 months | none | 48 | 290 | 0.16 | 90924 | high | normal | normal | - |
| 6 | male | 56 | biopsy | headache and dizziness | both | no | no | no | 3 years | 2 months | 123 | 538 | 0.23 | 388 | normal | normal | normal | - |
| 7 | male | 42 | biopsy | visual abnormalities, cognitive impairment | supratentorial | no | no | no | 7 days (initial manifestation) | none | 51 | 664 | 0.08 | 48427 | normal | normal | normal | - |
| 8 | female | 37 | biopsy | motor disorders, visual abnormalities | both | yes | no | no | 3 months | 2 months | 150 | 444 | 0.34 | 41 | high | normal | normal |  |
| 9 | male | 39 | biopsy | motor disorders, visual abnormalities | supratentorial | no | no | no | 5 days (initial manifestation) | 2 days | 35 | 719 | 0.05 | 50137 | normal | normal | normal | - |
| 10 | male | 26 | biopsy | visual abnormalities | supratentorial | no | no | no | 3 months | 3 months | 338 | 1611 | 0.21 | 152 | high | normal | normal | - |
| 11 | male | 59 | biopsy | motor disorders | both | no | no | yes | 2 months | 2 months | 92 | 291 | 0.32 | 46 | - | - | - | - |
| 12 | male | 36 | biopsy | motor disorders, visual abnormalities | infratentorial | no | no | no | 20 days (initial manifestation) | 13 days | 308 | 788 | 0.39 | 10811 | high | normal | normal | positive |
| 13 | male | 49 | biopsy | motor disorders, speech disorder,cognitive impairment | supratentorial | yes | no | yes | 2 months (initial manifestation) | 38 days | 60 | 1246 | 0.05 | 32 | normal | normal | normal | - |
| 14 | male | 30 | biopsy | headache and dizziness | infratentorial | no | no | no | 7 days | 8 days | 32 | 947 | 0.03 | 1983 | normal | normal | normal | - |
| 15 | male | 22 | biopsy | headache and dizziness, fever | supratentorial | yes | yes | no | 3 months | 2 months | 93 | 1414 | 0.07 | <20 | high | low | normal | positive |
| 16 | male | 33 | biopsy | speech disorder, cognitive impairment, headache and dizziness, fever | both | yes | no | no | 5 years | 2.5 months | 252 | 574 | 0.44 | 94 | high | normal | normal | negtive |
| 17 | male | 32 | biopsy | visual abnormalities, cognitive impairment,headache and dizziness | supratentorial | no | no | no | 2 months | 2 months | 242 | 1195 | 0.20 | 551 | normal | normal | normal | - |
| 18 | male | 31 | surgery | motor disorders, headache and dizziness | supratentorial | yes | yes | no | 3 months | 3 months | 325 | 1141 | 0.28 | 158 | high | normal | normal | positive |
| 19 | male | 53 | surgery | motor disorders, headache and dizziness | supratentorial | no | yes | yes | 9 days (initial manifestation) | none | 242 | 54.17 | 0.24 | 92200 | - | - | - | - |

NOTE. PML = Progressive multifocal leukoencephalopathy, HIV = human immunodeficiency virus, ART = active antiretroviral therapy, CSF = cerebrospinal fluid, mNGS = metagenomic next-generation sequencing.
