# Supplementary material for: The boundaries between PML and PML-IRIS: difficult to define, pathology may predict
Source: Front Cell Infect Microbiol. 2025 Jun 27;15:1607428. doi: 10.3389/fcimb.2025.1607428 (PMC12245874; doi:10.3389/fcimb.2025.1607428)
Supplement: Supplementary file 2 [file Table2.docx]

**Supplementary Table 2. the pathological features of PML**

| **NO.** | **Degree of inflammation** | **Active/Chronic inflammation** | **gitter cell infiltration** | **plasma cell infiltration** | **neutrophil infiltration** | **atypical astrocyte cells** | **Perivascular inflammatory infiltration** | **neuronal degeneration** | **mitotic figures** | **SV40 staining** | **JCV PCR** | **lesion tissues mNGS** | **co-pathogens in mNGS** |
| --- | --- | --- | --- | --- | --- | --- | --- | --- | --- | --- | --- | --- | --- |
| 1 | moderate to severe | chronic | none or less | yes | none or less | yes | lymphocytes and plasma cells | - | pathological mitotic figures | positive | positive | positive | None |
| 2 | moderate to severe | active | none or less | no | none or less | yes | none or less | - | no | positive | positive | positive | Streptococcu pneumoniae |
| 3 | moderate to severe | active | none or less | no | none or less | no | lymphocytes and plasma cells | yes | no | - | positive | positive | None |
| 4 | moderate to severe | active | none or less | yes | none or less | yes | lymphocytes and plasma cells | - | Creutzfeldt cells | positive | positive | positive | EBV |
| 5 | mild | chronic | more | no | none or less | yes | none or less | - | no | - | positive | positive | None |
| 6 | mild | active | more | yes | none or less | no | lymphocytes and plasma cells | - | no | positive | - | positive | EBV, MAC |
| 7 | mild | chronic | more | no | none or less | yes | gitter cells | no | no | positive | positive | positive | None |
| 8 | moderate to severe | chronic | none or less | yes | none or less | no | lymphocytes and plasma cells | no | no | positive | positive | positive | None |
| 9 | mild | chronic | more | no | none or less | no | gitter cells | - | no | positive | positive | - | - |
| 10 | moderate to severe | active | more | yes | more | no | lymphocytes and plasma cells | no | Creutzfeldt cells | positive | positive | positive | EBV |
| 11 | mild | chronic | more | no | none or less | yes | none or less | - | no | positive | - | positive | None |
| 12 | moderate to severe | active | more | no | none or less | no | none or less | - | no | positive | - | positive | None |
| 13 | moderate to severe | chronic | more | yes | more | no | lymphocytes and plasma cells | yes | Creutzfeldt cells | positive | positive | - | - |
| 14 | mild | chronic | none or less | no | none or less | no | none or less | no | no | positive | - | positive | None |
| 15 | moderate to severe | active | none or less | yes | more | no | lymphocytes and plasma cells | yes | no | positive | - | positive | None |
| 16 | moderate to severe | chronic | none or less | yes | none or less | no | lymphocytes and plasma cells | yes | no | positive | - | positive | None |
| 17 | moderate to severe | active | none or less | yes | more | no | lymphocytes and plasma cells | no | no | positive | - | positive | None |
| 18 | moderate to severe | active | none or less | yes | none or less | no | lymphocytes and plasma cells | no | no | positive | - | positive | None |
| 19 | mild | chronic | none or less | no | none or less | no | none or less | yes | no | positive | - | positive | None |

NOTE. PML = Progressive multifocal leukoencephalopathy, JCV = John Cunningham virus, mNGS = metagenomic next-generation sequencing, EBV = Epstein-Barr virus, MAC = Mycobacterium avium complex.
