# Supplementary material for: The boundaries between PML and PML-IRIS: difficult to define, pathology may predict
Source: Front Cell Infect Microbiol. 2025 Jun 27;15:1607428. doi: 10.3389/fcimb.2025.1607428 (PMC12245874; doi:10.3389/fcimb.2025.1607428)
Supplement: Supplementary file 3 [file Table3.docx]

**Supplementary Table 3. Clinicopathological Features of PML in the ART Era.**

|  | | **ART ( < 4 weeks)**  **N = 8** | **ART (4-12 weeks)**  **N = 9** | **ART ( > 12 weeks)**  **N = 2** |
| --- | --- | --- | --- | --- |
| **Age(years)** | | 40.0±7.7 | 35.8±13.1 | 30.0±19.8 |
| **Median Age (years)** | | 37.5(30-53) | 32.0(22-59) | 30.0(16-44) |
| **blood CD4+ T-cell counts (cells/μl)** | | 115.0±106.3 | 197.1±95.1 | 394.0±530.3 |
| **blood CD8+ T-cell counts (cells/μl)** | | 726.2±398.0 | 859.1±481.8 | 701.0±530.3 |
| **blood HIV viral load (copies/ml)** | | 54277.0±50279.8 | 210.6±203.7 | 50927.5±19.8 |
| **CSF protein (mg/dl)** | | 44.7±15.9 | 51.1±23.0 | 69.6±45.8 |
| **CSF glucose (mmol/l)** | | 3.2±0.5 | 3.1±0.3 | 3.3±0.5 |
| **Mass effect (yes/no)** | | 1/7 | 2/7 | 1/1 |
| **Contrast enhancement (yes/no)** | | 1/7 | 4/5 | 1/1 |
| **Restricted diffusion (yes/no)** | | 2/6 | 1/8 | 0/2 |
| **Degree of inflammation** | mild | 5(26.3%) | 2(10.5%) | 0 |
|  | moderate to severe | 3(15.8%) | 7(36.8%) | 2(10.5%) |
| **Active/Chronic inflammation** | Active | 6(31.6%) | 3(15.8%) | 1(5.3%) |
|  | Chronic | 2(10.5%) | 6(31.6%) | 1(5.3%) |
| **Gitter cell infiltration** | none or less | 3(15.8%) | 6(31.6%) | 2(10.5%) |
|  | More | 5(26.3%) | 3(15.8%) | 0 |
| **Perivascular inflammatory infiltration** | lymphocytes and plasma cells | 2(10.5%) | 8(42.1%) | 1(5.3%) |
|  | less or gitter cells | 6(31.6%) | 1(5.3%) | 1(5.3%) |
| **Neutrophil infiltration** | none or less | 7(36.8%) | 6(31.6%) | 2(10.5%) |
|  | More | 1(5.3%) | 3(15.8%) | 0 |
| **Plasma cell infiltration** | Yes | 2(10.5%) | 7(36.8%) | 1(5.3%) |
|  | No | 6(31.6%) | 2(10.5%) | 1(5.3%) |

NOTE. PML = Progressive multifocal leukoencephalopathy, HIV = human immunodeficiency virus, ART = active antiretroviral therapy, CSF = cerebrospinal fluid.

Due to the limited number of cases, only statistical descriptions were performed.
