# Supplementary material for: The boundaries between PML and PML-IRIS: difficult to define, pathology may predict
Source: Front Cell Infect Microbiol. 2025 Jun 27;15:1607428. doi: 10.3389/fcimb.2025.1607428 (PMC12245874; doi:10.3389/fcimb.2025.1607428)
Supplement: Supplementary file 4 [file Table4.docx]

**Supplementary Table4. Clinical and Pathological Characteristics of PML and PML-IRIS**

|  | | **PML (N=15)** | **PML-IRIS (N=4)** |
| --- | --- | --- | --- |
| **Age(years)** | | 39.4±11.6 | 27.8±4.6 |
| **Median Age (years)** | | 37.0(16-59) | 28.5(22-32) |
| **The duration of ART (months)** | | 2.3±3.7 | 2.5±0.6 |
| **blood CD4+ T-cell counts (cells/μl)** | | 165.6±189.0 | 249.5±112.7 |
| **blood CD8+ T-cell counts (cells/μl)** | | 638.8±342.8 | 1340.3±215.7 |
| **blood HIV viral load (copies/ml)** | | 35807.0±48098.2 | 215.3±235.5 |
| **CSF protein (mg/dl)** | | 46.9±21.4 | 62.8±26.0 |
| **CSF glucose (mmol/l)** | | 3.2±0.4 | 3.0±0.2 |
| **Mass effect (yes/no)** | | 2/13 | 2/2 |
| **Contrast enhancement (yes/no)** | | 4/11 | 2/2 |
| **Restricted diffusion (yes/no)** | | 3/12 | 0/4 |
| **Degree of inflammation** | mild | 7(36.8%) | 0 |
|  | moderate to severe | 8(42.1%) | 4(21.1%) |
| **Active/Chronic inflammation** | Active | 10(52.7%) | 0 |
|  | Chronic | 5(26.3%) | 4(21.1%) |
| **Gitter cell infiltration** | none or less | 8(42.1%) | 3(15.8%) |
|  | More | 7(36.8%) | 1(5.3%) |
| **Perivascular inflammatory infiltration** | lymphocytes and plasma cells | 7(36.8%) | 4(21.1%) |
|  | less or gitter cells | 8(42.1%) | 0 |
| **Neutrophil infiltration** | none or less | 14(73.7%) | 1(5.3%) |
|  | More | 1(5.3%) | 3(15.8%) |
| **Plasma cell infiltration** | Yes | 6(31.6%) | 4(21.1%) |
|  | No | 9(47.4%) | 0 |

NOTE. PML = Progressive multifocal leukoencephalopathy, HIV = human immunodeficiency virus, ART = active antiretroviral therapy, CSF = cerebrospinal fluid, IRIS = immune reconstitution inflammatory syndrome.

Due to the limited number of cases, only statistical descriptions were performed.
